# Salivary biomarkers for the prognosis of oncological and infectious diseases: a systematic review

**DOI:** 10.3389/fdmed.2025.1662276

**Published:** 2025-09-29

**Authors:** Heber Isac Arbildo-Vega, Saurav Panda, Fredy Hugo Cruzado-Oliva, Hernán Vásquez-Rodrigo, Rubén Aguirre-Ipenza, Joan Manuel Meza-Málaga, Sara Antonieta Luján-Valencia, Eduardo Luján-Urviola, Carlos Alberto Farje-Gallardo, Tania Belú Castillo-Cornock, Katherine Serquen-Olano, Tania Padilla-Cáceres, Luz Caballero-Apaza, Franz Tito Coronel-Zubiate

**Affiliations:** ^1^ Department of General Dentistry, Dentistry School, Universidad San Martín de Porres, Chiclayo, Peru; ^2^ Department of Human Medicine, School of Human Medicine, Universidad San Martín de Porres, Chiclayo, Peru; ^3^ Posgraduate School, Universidad Nacional Toribio Rodríguez de Mendoza de Amazonas, Chachapoyas, Peru; ^4^ Department of Periodontics and Oral Implantology, Siksha ‘O’ Anusandhan Univeristy, Bhubaneswar, India; ^5^ Department of Stomatology, School of Stomatology, Universidad Nacional de Trujillo, Trujillo, Peru; ^6^ Department of Dentistry, Dentistry School, Universidad Norbert Wiener, Lima, Peru; ^7^ Faculty of Health Sciences, Universidad Continental, Lima, Peru; ^8^ Faculty of Medicine, Medicine School, Universidad Católica de Santa María, Arequipa, Peru; ^9^ Postgraduate School, Universidad Católica de Santa María, Arequipa, Peru; ^10^ Faculty of Dentistry, Dentistry School, Universidad Católica de Santa María, Arequipa, Peru; ^11^ Faculty of Dentistry, Universidad Andina Néstor Cáceres Velásquez, Juliaca, Peru; ^12^ Faculty of Health Sciences, Stomatology School, Universidad Nacional Toribio Rodríguez de Mendoza de Amazonas, Chachapoyas, Peru; ^13^ Faculty of Health Sciences, Stomatology School, Universidad Señor de Sipán, Chiclayo, Peru; ^14^ Department of General Dentistry, Dentistry School, Universidad Nacional del Altiplano, Puno, Peru; ^15^ Department of Nursing, School of Nursing, Universidad Nacional del Altiplano, Puno, Peru

**Keywords:** salivary biomarker, interleukin, oncology, infectious diseases, prognosis, systematic review

## Abstract

**Objective:**

To determine the salivary biomarkers that are used in the prognosis of oncological and infectious diseases.

**Materials and methods:**

A bibliographic search was carried out until July 2025, in the biomedical databases: PubMed, Cochrane Library, Scopus, EMBASE, Web of Science (WoS), Scielo, Science Direct and Google Scholar. Studies that were clinical trials, which reported the use of salivary biomarkers for the prognosis of oncological and infectious diseases, without time and language limits, were included. The Cochrane Handbook of Systematic Reviews of Interventions was used to assess the risk of bias of the included studies.

**Results:**

The preliminary search yielded a total of 189 articles, discarding those that did not meet the selection criteria, leaving only 16 articles for qualitative synthesis. These studies reported that the most widely used salivary biomarkers in the prognosis of oncological and infectious diseases are cortisol and interleukins.

**Conclusions:**

Salivary biomarkers, especially cortisol and key interleukins, demonstrate potential as non-invasive tools for the prognostic assessment and monitoring of oncological and infectious diseases. Further standardization and clinical validation are needed to support their integration into routine practice.

**Systematic Review Registration:**

https://www.crd.york.ac.uk/PROSPERO/view/CRD42021260764, PROSPERO CRD42021260764.

## 1 Introduction

The salivary glands in humans secrete a biological fluid called saliva. health requires a variety of organic and inorganic materials, proteins, immunoglobulins, cytokines (1). This fluid has distinct advantages over other biological samples and is being utilized for diagnostic purposes (2).

The use of salivary cytokines as diagnostic biomarkers for a range of oral disorders, including dental caries and oral cancer, is supported by molecular analysis conducted in multiple investigations. Salivary cytokines’ role in systemic inflammatory processes has also been linked to a number of general illnesses, suggesting that they may be helpful in the diagnosis and prognosis of systemic diseases (3).

The focus of research on salivary diagnosis has changed over time, moving away from examining the relationships between specific biomarkers and systemic or local illnesses (4, 5). More recent studies have included extremely sensitive devices that can measure many cytokines in little amounts of saliva due to advancements in scientific technologies (6). Salivary cytokines’ potential as indicators in the treatment of oral illnesses has been highlighted by a number of reviews that have systematized the evidence that is currently available (7, 8).

Infectious diseases—including HIV/AIDS, tuberculosis, viral hepatitis, and respiratory viral infections—represent a substantial global health challenge and often trigger systemic inflammatory responses mediated by cytokines and stress hormones (9). Salivary biomarkers such as IL-1β, IL-6, IL-8, TNF-α, and cortisol have been correlated with disease progression and immune activation in these conditions, enabling non-invasive monitoring (10).

Given that infectious and oncological diseases represent two of the most prevalent and burdensome global health challenges, this review focused specifically on these categories. Both types of diseases are known to induce systemic inflammatory and neuroendocrine alterations, which in turn affect the expression of cytokines and stress-related hormones such as cortisol in saliva (2). A comparative overview of salivary biomarkers across these two disease types may reveal shared immune mechanisms, as well as condition-specific biomarker profiles, with implications for early diagnosis, prognosis, and monitoring using non-invasive techniques. This integrated approach reflects current trends in predictive, preventive, and personalized medicine and addresses a gap in previous reviews, which often analyze these conditions separately.

The identification of salivary cytokines in relation to a range of clinical conditions, such as psychiatric disorders (11), rheumatoid diseases like Sjögren's syndrome (12, 13), cystic fibrosis (14), sleep apnea (15), oncological, and infectious diseases (2), has been the subject of numerous studies. Salivary biomarkers have not yet been proven to be useful in the prognosis of systemic disorders, nevertheless. Thus, reviewing the potential of salivary biomarkers in infectious and oncological diseases in terms of their prediction was the goal of the current study.

## 2 Materials and methods

### 2.1 Protocol and registration

All authors helped define the protocol for this systematic review, which was developed according to the Preferred Reporting Items for Systematic Reviews and Meta-Analysis Protocols (PRISMA-P) (16). In addition, this protocol was registered with the number CRD42021260764 (https://www.crd.york.ac.uk/PROSPERO/view/CRD42021260764) in the Prospective International Registry of Systematic Reviews (PROSPERO) (17).

To prepare and structure this review, the focused question was formulated using the PICO format (population, intervention, comparison and outcomes) as detailed below:

• Population: People with oncological and infectious diseases
• Intervention: Treatment of oncological and infectious disease
• Comparison: No treatment, placebo or conventional treatment of oncological and infectious disease
• Outcomes: Salivary biomarker

### 2.2 Focused question (PICO)

What are the salivary biomarkers available as a prognosis for oncological and infectious diseases?

### 2.3 Search and selection of studies

For the present systematic review, a bibliographic search was carried out in 8 electronic databases (Pubmed, Cochrane Library, Scopus, EMBASE, Web of Science (WoS), Scielo, Sciencedirect and Google Scholar) until July 2025; combining keywords and subject titles according to the thesaurus of each database: “saliv*”, “cytokine”, “biomaker”, “interleukin”, “neoplasms”, “HIV”, “Epstein -Barr virus”, “EBV”, “tuberculosis” and “clinical trial”. The search strategies of each of the databases are found in Table 1.

**Table 1:** Search strategies for each database.

Database	Search strategy	Number of study
Pubmed	(saliv*) AND {[(Cytokine) OR Biomaker] OR Interleukin} AND (((((neoplasms) OR HIV) OR “Epstein -Barr virus”) OR EBV) OR Tuberculosis) AND (“clinical trial”)	46
Cochrane library	#1 MeSH descriptor: (Saliva) explode all trees #2 (saliv*) (Word variations have been searched) #3 #1 OR #2 #4 MeSH descriptor: (Cytokines) explode all trees #5 MeSH descriptor: (Biomarkers) explode all trees #6 MeSH descriptor: (Interleukins) explode all trees #7 (cytokine) OR (biomarker) OR (interleukin) (Word variations have been searched) #8 #4 OR #5 OR #6 OR #7 #9 MeSH descriptor: (Neoplasms) explode all trees #10 MeSH descriptor: (HIV) explode all trees #11 MeSH descriptor: (Herpesvirus 4, Human) explode all trees #12 MeSH descriptor: (Tuberculosis) explode all trees #13 (neoplasm) OR (HIV) OR (Epstein Barr Virus) OR (EBV) OR (Tuberculosis) (Word variations have been searched) #14 #9 OR #10 OR #11 OR #12 OR #13 #15 #3 AND #8 AND #14	72
Scopus	(TITLE-ABS-KEY (saliv*)) AND (TITLE-ABS-KEY (cytokines) OR TITLE-ABS-KEY (biomarkers) OR TITLE-ABS-KEY (interleukin)) AND (TITLE-ABS-KEY (neoplasm) OR TITLE-ABS-KEY (HIV) OR TITLE-ABS-KEY (“Epstein Barr Virus”) OR TITLE-ABS-KEY (EBV) OR TITLE-ABS-KEY (Tuberculosis)) AND (TITLE-ABS-KEY (“clinical trial”)) AND (LIMIT-TO (DOCTYPE, “ar”)) AND (LIMIT-TO (SUBJAREA, “DENT”)) AND (LIMIT-TO (SRCTYPE, “j”))	64
EMBASE	(saliv*:ti,ab,kw) AND (cytokines:ti,ab,kw OR biomarkers:ti,ab,kw OR interleukin:ti,ab,kw) AND (neoplasm:ti,ab,kw OR HIV:ti,ab,kw OR ‘Epstein Barr Virus’:ti,ab,kw OR EBV:ti,ab,kw OR tuberculosis:ti,ab,kw) AND (‘clinical trial':ti,ab,kw)	41
Web of Science	(TS = (saliv*)) AND (TS = (cytokines) OR (biomarkers) OR (interleukin)) AND (TS = (neoplasm) OR (HIV) OR (“Epstein Barr Virus”) OR (EBV) OR (tuberculosis)) AND (TS = (“clinical trial”))	49
Scielo	(saliv*) AND (((Cytokine) OR Biomaker) OR Interleukin) AND (((((neoplasms) OR HIV) OR “Epstein -Barr virus”) OR EBV) OR Tuberculosis) AND (“clinical trial”)	3
Sciencedirect	(saliv*) AND (((Cytokine) OR Biomaker) OR Interleukin) AND (((((neoplasms) OR HIV) OR “Epstein -Barr virus”) OR EBV) OR Tuberculosis) AND (“clinical trial”)	4
Google scholar	“saliv*” + “Cytokine” OR “Biomaker” OR “Interleukin” + “neoplasms” OR “HIV” OR “Epstein -Barr virus” OR “EBV” OR “Tuberculosis” + “clinical trial”	78

All search strategies were constructed using database-specific controlled vocabularies (e.g., MeSH in PubMed, Emtree in EMBASE, DeCS in SciELO). These terms cover related concepts hierarchically, allowing inclusion of broader biomarker classes—such as RNAs and other salivary components—as well as diverse neoplasm subtypes, even when not explicitly mentioned in the query.

Additional relevant literature was also included through manual review of the reference lists of the finally selected articles.

The search of electronic databases was performed independently by two reviewers (HA and SP), and the inclusion of studies was determined based on predefined criteria: randomised clinical trials (RCTs) published in English, with no restriction on the date of publication, and reporting on the use of salivary biomarkers for prognostic purposes in oncological and/or infectious diseases.

### 2.4 Data extraction

Data extraction from each eligible study was performed using a structured template that included key variables such as year of publication, authorship, country of origin, study title, sample size, gender distribution, associated systemic condition, mean age, group allocation, salivary biomarkers assessed, analytical methods used, presence of additional biomarkers, follow-up duration, and conclusion. Two independent reviewers (FCO and HV) performed the data extraction, and any discrepancies were resolved by consensus with a third reviewer (FCZ).

### 2.5 Risk of bias (RoB) assessment

Using the Cochrane Risk of Bias 2.0 tool, two calibrated reviewers (RA and JM) separately assessed the risk of bias (RoB) of the included studies (18), and a high level of inter-reviewer agreement was observed (k = 0.98). Randomisation procedure, deviations from planned interventions, lack of outcome data, outcome assessment, and selection of reported outcomes are the five categories in which this instrument evaluates randomised clinical trials. Studies are classified as low risk of bias, moderate concerns or high risk of bias according to these factors.

### 2.6 Analysis of results

A qualitative evaluation of the included studies’ findings was the sole focus of the synthesis. Furthermore, GRADE analysis was used to evaluate the evidence's certainty using the GRADEPro GDT guideline development tool, which was created by McMaster University and Evidence Prime Inc. in Canada.

## 3 Results

### 3.1 Selection of studies

**Figure 1:**
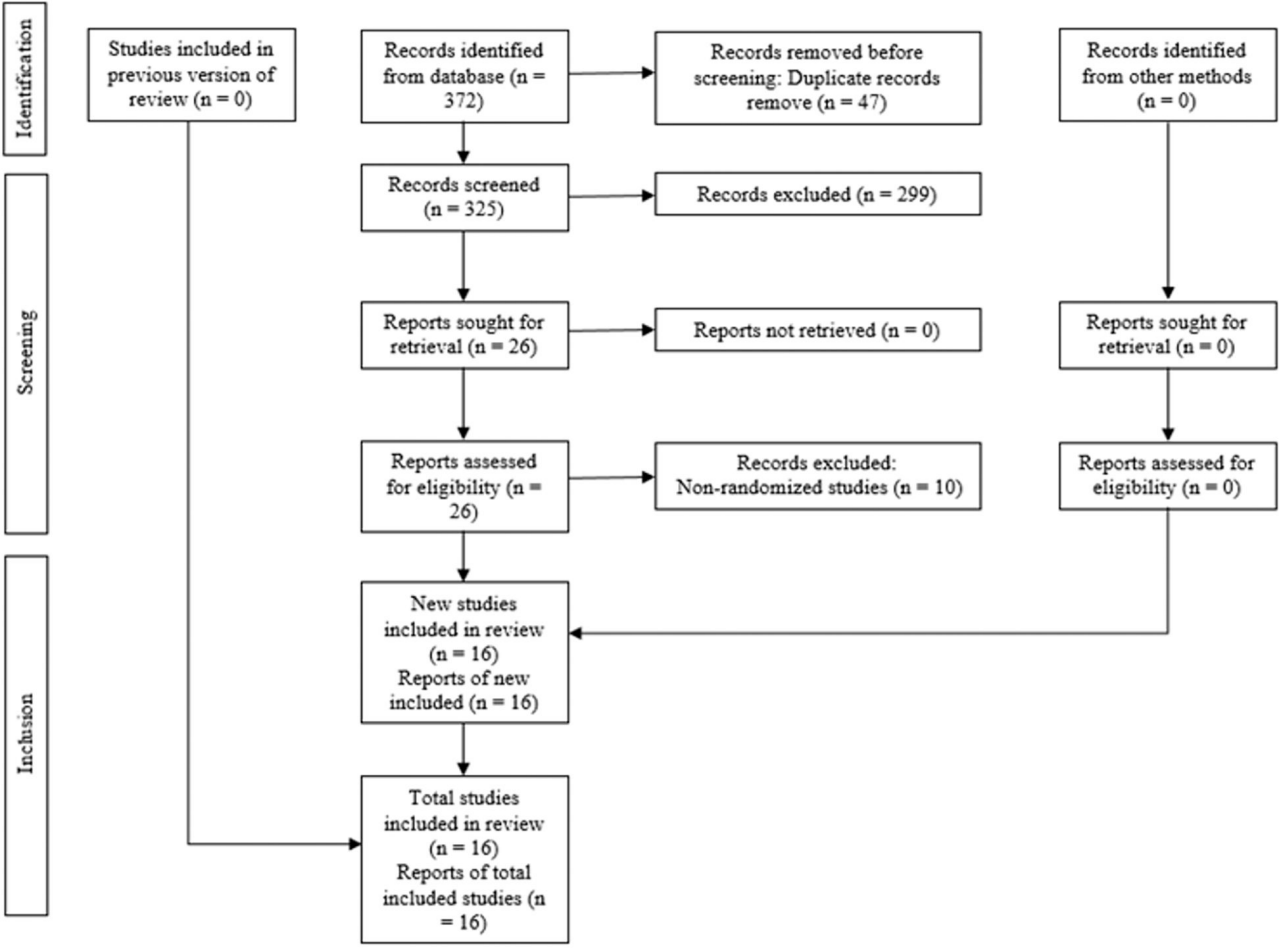
PRISMA flowchart showing the process of inclusion and exclusion of studies in the systematic review.

A comprehensive manual and electronic search initially identified 372 records, of which 47 duplicates were removed (Figure 1). After reviewing the titles and abstracts, 26 full-text articles were considered potentially eligible and retrieved for detailed assessment. Of these, 10 did not meet the inclusion criteria and were excluded. Consequently, 16 randomised controlled trials were considered eligible and included in the qualitative synthesis. The specific reasons for exclusion are indicated in Table 2.

**Table 2:** Reason for exclusion of the studies.

Authors	Exclusion reason
Deepthi et al. (19), Carvalho et al. (20), Jacobs et al. (21), Pels (22), Sharma et al. (23), Carlson et al. (24), Small et al. (25), Rhodus et al. (26), Abdel Fattah et al. (27), Ucciferri et al. (28)	Non-randomized studies

### 3.2 Characteristics of included studies

Overall, 16 RCTs (29–44) with publication years ranging from 2008 to 2024 were included. The countries where the studies were conducted were: United States (31–35, 37, 39, 43, 44), South Africa (36), Brazil (29, 30, 38, 39), United Kingdom (41) and Venezuela (41) (Table 3).

**Table 3:** Characteristic of included studies.

Author	Year	Country	Number of patients (M/W)	Target condition	Mean age	Groups	Patients per group	Salivary biomarker	Salivary sample analysis method	Other biomarkers	Follow-up	Conclusions
Cancer												
Pereira et al. (29)	2024	Brazil	52 (31/21)	Head and neck cancer	57.08 ± 11.2	FITOPROT + PBMT	25	IL-1, TNFα, IL-6, IL-8 e IL-12p70 e IL-10	The cytometric bead array human infammatory cytokines kit (BD Biosciences, San Jose, CA, USA)	Nitrite	6 weeks	FITOPROT + PBMT could be associated with the balance of nitrites and cytokines.
						PBMT	27					
Martins et al. (30)	2021	Brazil	45 (41/7)	Head and neck cancer	59.7 ± 12.53	PBMT	25	IL-6, IL-8, IL-10, IL-12p70, IL-1β e TNF-α	Cytometric bead array, measured by a BD FACSCanto II flow cytometer (BD Bio sciences, San Jose, CA)	Nitrite	30 days	PBMT promoted an increase in the concentration of IL-12p70, TNF-α and IL-10
						POCP	23					
Hoyt et al. (31)	2021	United State	27 (27/0)	Testicular cancer	27.7 ± 4.2	GET	12	Cortisol	Salivette collection tubes (Sarstedt, Inc., Newton, NC)	PCR, IL-6, IL-1ra, TNFαRII, VEGF	8 weeks	There was a decrease in cortisol production from baseline to post-intervention in those receiving GET
						ISP	15					
Penedo et al. (32)	2021	United State	192 (192/0)	Prostate cancer (stage III)	68.84 ± 8.87	CBSM	95	Cortisol	Salivettes® (Sarstedt AG & Co.)	TNFα, PCR, IL-6, IL-8, IL-10	1 year	Patients in both CBSM and control groups showed decreases in IL-10, IL-8, and TNF-α from baseline to 6 months. However, these markers generally showed a rebound increase from 6 to 12 months
						Health promotion	97					
Basak et al. (33)	2020	United State	25 (24/1)	Oral cancer	57.28 ± 11.9	Placebo	12	IFN-γ, IL-10, IL-12p70, IL-1β, IL-2, IL-8, TNF-α, GM-CSF, IL-13, IL-4	NR	NR	1 day	Decreases salivary biomarkers (IL-1β, IL-6 and IL-8) post treatment, resulting in a decrease in bacteroids and anti-inflammatory cytokines in patients with oral cancer undergoing treatment with APG-157
						APG-157	13					
Lengacher et al. (35)	2019	United State	322 (0/322)	Breast cancer	56.6 ± 9.7	MBSR	167	Cortisol, IL-6	Salimetrics High Sensitivity Salivary Cortisol Enzyme Immunoassay Kit and IL-6 High sensitivity ELISA kit (ABCom)	NR	6 weeks	Post-treatment reduction in salivary cortisol and IL-6 biomarkers. Resulting in stress reduction in breast cancer patients who undergo the relaxation program.
						Usual care	155					
Campo et al. (37)	2015	United State	63 (0/63)	Cancer	67 ± 7.15	Tai Chi Chih	32	Cortisol	Salivettes® (Sarstedt AG & Co.)	IL-12, IL-6, TNF-α, IL-10, IL-4	12 weeks	Decrease in salivary cortisol biomarkers post-treatment, resulting in a decrease in risk factors for chronic disease in breast cancer patients who undergo the Tai Chi program.
						Health education control	31					
Oton-Leite et al. (38)	2015	Brazil	25 (21/4)	Head and neck cancer	NR	Laser	12	TNF-α, IL-1β, IL-10, EGF, FGF, VEGF, MMP2/TIMP2, MMP9/TIMP2, IL-6	Quantitative Sandwich ELISA Kits and IL-6 High Sensitivity Kit	NR	7 weeks	There was a trend for reduced levels of IL-1β, TNF-α, IL-10, in the laser group compared to the control, however, no differences were found.
						Control	13					
Silva et al. (39)	2015	Brazil	25 (12/13)	Cancer	36.7	Laser	11	TNF-α, IL-1β, IL-10, TGF-α, EGF, FGF, VEGF, MMP2/TIMP2, MMP9/TIMP2, IL-6	Quantitative sandwich enzyme linked immunosorbent assay (ELISA) kits (DuoSet, R&D Systems, Minneapolis, MN, USA) and IL-6 Quantikine ELISA High-Sensitivity kit	NR	21 days	Salivary biomarkers such as IL-6, TNF-α, IL-1β, IL-10, MMP-2/TIMP2 increase post-treatment. Resulting in decreased oral mucositis in transplant patients who undergo low intensity laser treatment.
						Control	14					
Bower et al. (40)	2014	United State	31 (0/31)	Breast cancer	54 ± 5.4	Iyengar yoga	16	Cortisol	Salivettes® (Sarstedt, Inc.)	TNF-α, IL-1β, IL-6, CRP	12 weeks	Post-treatment cortisol alteration is not evidenced.
						Health education control	15					
Saxton et al. (41)	2014	United Kingdom	85 (0/85)	Breast cancer	55.5	Lyfestile intervention	44	Cortisol	Salivettes® (Sarstedt, Leicester, UK)	IL-6, TNF-α	6 months	Increase in salivary cortisol biomarkers post treatment, resulting in the reduction of depressive symptoms in patients with breast cancer submitted to the exercise program and healthy eating.
						Control	41					
Morales-Rojas et al. (42)	2012	Venezuela	42	Acute lymphoblastic leukemia	NR	Methotrexate	21	TNF-α, IL-1, IL-6	ELISA for which commercial Kits (R&D Systems; TECHNE Corpotaration, Minneapolis, MN, USA)	NR	4 days	Increase the salivary biomarkers IL-6, TNF-α, resulting in being able to prevent oral mucositis in patients taking Methotrexate.
						Control	21					
Nelson et al. (44)	2008	United State	50 (0/50)	Cervical cancer	49.15	PTC	27	Cortisol, DHEA	Salivettes® (Sarstedt)	IL-10, INF-γ, IL-5	4 months	Decrease in salivary cortisol biomarkers post-treatment, resulting in decreased stress in cervical cancer patients undergoing psychosocial counseling treatment.
						Usual care	23					
HIV												
Rubin et al. (34)	2020	United State	81 (45/36)	HIV	34.49	Low-dose hydrocortisone	81	Cortisol, IL-6, IL-8, IL-1β, TNF-α, CRP, IP-10, MCP-1, MIG, MMP-9, MMP-1	ELISA kit by Salimetrics and MILLIPLEX MAP (Millipore, Billerica, MA)	NR	4 h	They increase salivary biomarkers, resulting in memory improvement in HIV patients after hydrocortisone treatment.
						Placebo	81					
Dudgeon et al. (43)	2010	United State	38 (38/0)	HIV	43.9	MOD	14	Cortisol	Salivettes® (Sarstedt) and ELISA	IL-6, IFG-1, IGFBP-3, GH, TNF-α, IL-1β	3 days	The individual sessions of both low-intensity and moderate-intensity exercise can alter circulating anabolic and catabolic factors in HIV-infected men.
						LOW	11					
						Control	13					
Tuberculosis												
Shenje et al. (36)	2018	South Africa	14	Pericardial tuberculosis	35.9 ± 12.5	Prednisone	5	IFN-γ, IL-1α, IL-1β, IL-6, IL-10, IL-12p40, TNF-α, IL-8, IP-10	Customized Milliplex™ kits (HCYTOMAG-60K, Millipore, St Charles, MO, USA)	NR	1 day	They decrease the salivary biomarkers IL-1β, IL-8 and increase IL-6, resulting in being able to prevent inflammation in patients with pericardial tuberculosis taking prednisolone.
						Placebo	9					

NR, not registrable; RCT, randomized clinical trial; PBMT, photobiomodulation therapy; POCP, preventive oral care program; GET, goal-focused emotion-regulation therapy; ISP, individual supportive psychotherapy; CBSM, cognitive behavioral stress management; MBSR, mindfulness-based stress reduction; PTC, psychosocial telephone counseling; MOD, moderate-intensity exercise; LOW, low-intensity exercise; IL, interleukin; TNF, tumor necrosis factor; MMP, matrix metalloproteinases; INF, interferon.

The studies in Table 3 are listed in reverse chronological order by year of publication to highlight the temporal progression of biomarker research in prognostic settings.

The number of patients in all studies ranged from 14 to 322. Systematic diseases or conditions in all studies were: cancer (29–33, 35, 37–42, 44), HIV (34, 43) and tuberculosis (46); having as treatments drugs (33, 34, 36, 42), relaxation exercises and stress reduction (31, 32, 35, 37, 40, 41, 43, 44) and laser (29, 30, 38, 39). The mean age was reported in 14 studies (29, 31–37, 39–41, 43, 44) which ranged between 34.49 and 67 years. Follow-up time in all studies ranged from 4 h to 6 months. The most used salivary biomarkers were cortisol and interleukins (Table 3).

### 3.3 Risk of bias analysis of studies

**Figure 2:**
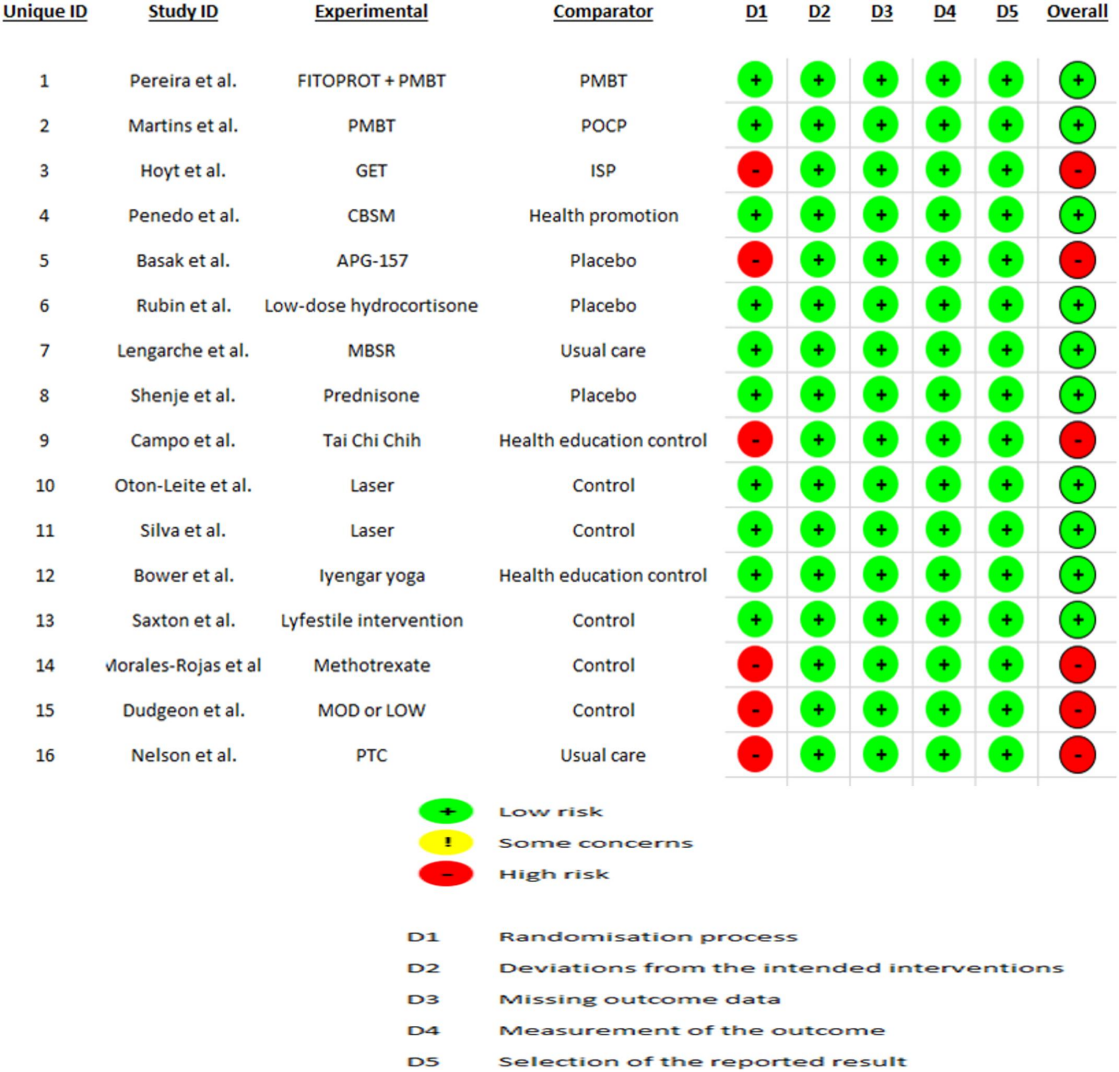
Risk of bias analysis of included studies.

Ten studies (29, 30, 32, 34–36, 38–41) were at low risk of bias, and 6 studies (31, 33, 37, 42–44) were at high risk of bias (Figure 2).

The most frequent sources of bias were related to blinding of outcome assessors, incomplete outcome data, and lack of pre-specified protocols. Studies using biomarker assays without standardized saliva collection protocols showed increased detection bias. Among the six studies rated with high risk of bias, most reported positive findings, raising the possibility of publication or reporting bias.

### 3.4 GRADE analysis

The GRADE assessment indicated low overall certainty of the evidence, primarily due to serious concerns related to risk of bias and inconsistency. Several studies lacked robust randomization methods or blinded outcome assessments. Inconsistencies in assay methods and outcome definitions contributed to heterogeneity in findings. Although indirectness and imprecision were not serious issues, the lack of external validation and variability in sample sizes limited the confidence in the clinical applicability of the findings (see Table 4).

**Table 4:** GRADE analysis of included studies.

Certainty assessment							Certainty
№ of studies	Study design	Risk of bias	Inconsistency	Indirectness	Imprecision	Other considerations	
16	randomized trials	serious	serious	not serious	not serious	none	⊕⊕○○ Low

This table presents the overall GRADE assessment for the certainty of evidence across the included randomized controlled trials. The rating was downgraded due to serious concerns regarding risk of bias and inconsistency.

**Symbols used:** ⊕ = high certainty, ⊕⊕ = moderate certainty, ⊕⊕○○ = low certainty, ⊕○○○ = very low certainty.

### 3.5 Summary of main findings

The 16 included randomized controlled trials explored the use of various salivary biomarkers for monitoring oncological and infectious diseases, predominantly focusing on cytokines such as IL-6, IL-1β, IL-8, TNF-α, cortisol, and CRP. These biomarkers were evaluated in contexts ranging from cancer-related fatigue and stress responses to infectious disease progression in HIV and tuberculosis patients.

Among the studies, interleukins (particularly IL-6 and IL-1β) were frequently used to reflect proinflammatory activity. Cortisol levels were studied in relation to psychoneuroimmunological responses to disease and stress interventions, such as relaxation therapy. While methodological approaches varied, common findings included the potential diagnostic and prognostic value of these biomarkers, despite challenges like salivary variability, sample handling, and lack of standardization across assays.

To aid interpretation, Table 5 presents a comprehensive overview of each major biomarker's biological relevance, clinical utility, methodological limitations, and proposed directions for future research. This synthesis aims to guide researchers and clinicians in prioritizing salivary biomarker candidates and designing standardized protocols for future applications.

**Table 5:** Summary of salivary biomarkers for oncological and infectious diseases.

Biomarker	Disease type	Biological significance	Clinical utility	Methodological limitations	Future research directions
IL-6	Both (Oncological + Infectious)	Regulates inflammation; dual role (pro/anti-inflammatory)	Monitor disease activity, response to therapy	Varies by assay; fluctuations can be transient	Standardized assays and longitudinal studies
IL-1β	Both (Oncological + Infectious)	Key proinflammatory cytokine; involved in cancer invasiveness and infection response	Early diagnosis; progression marker	High interindividual variability	Define normal ranges by disease stage
IL-8	Both (Oncological + Infectious)	Recruits neutrophils; associated with tumor growth and infection	Infection marker, linked with angiogenesis cancer	Sensitive to salivary flow and sample handling	Combine with microbiome data for validation
TNF-α	Both (Oncological + Infectious)	Promotes apoptosis and systemic inflammation	Potential as a severity indicator	Low levels in early stages; matrix effect in saliva	Explore time-course profiles with treatment
Cortisol	Both (Oncological + Infectious)	Stress hormone; modulates immune response	Associated with prognosis, particularly in cancer	Affected by diurnal variation, stress	Assess in conjunction with cytokines
CRP (C-reactive protein)	Infectious (mainly)	Acute-phase protein; reflects systemic inflammation	Short-term monitoring of infectious flare-ups	Not consistently detectable in saliva	Development of ultrasensitive salivary CRP assays
IFN-γ	Infectious	Activates macrophages; antiviral responses	Potential for detecting viral infectious (e.g., COVID-19)	Often low concentration in saliva	Validate in viral infection cohorts
VEGF	Oncological	Promotes angiogenesis and tumor vascularization	Potential biomarker for tumor aggressiveness	High variability across cancers	Integrate with imaging biomarkers

This table summarizes key salivary biomarkers identified across the included randomized controlled trials. The information presented is based on narrative synthesis of the findings, highlighting the biological relevance, clinical applicability, and research gaps associated with each biomarker in oncological and infectious disease contexts.

## 4 Discussion

This systematic review evaluated the prognostic value of salivary biomarkers in oncological and infectious diseases, based on evidence from 16 randomized controlled trials. The findings demonstrate that certain salivary biomarkers—most notably cortisol and specific interleukins—exhibited measurable changes in response to therapeutic interventions, suggesting their potential utility in disease monitoring. In the following sections, we analyze the biological significance, clinical implications, and methodological limitations of these biomarkers, as well as directions for future research.

### 4.1 Overview of findings and clinical relevance

This systematic review included 16 randomized controlled trials evaluating the prognostic utility of salivary biomarkers in oncological and infectious diseases. The most commonly reported biomarkers were interleukins (especially IL-1β, IL-6, IL-8, and IL-10) and cortisol, which were frequently elevated in patients with systemic inflammatory conditions and reduced following therapeutic interventions. These findings highlight the growing potential of saliva as a non-invasive medium for monitoring disease progression and treatment response in diverse clinical scenarios (45, 46).

The clinical relevance of salivary biomarkers lies in their ability to reflect not only local but also systemic pathophysiological changes, offering a promising adjunct to traditional diagnostic and monitoring methods. For example, IL-6 is known to exhibit both pro- and anti-inflammatory properties and is actively involved in B and T cell differentiation, while IL-8 contributes to neutrophil recruitment and activation in acute inflammation (35). Cortisol, a key stress hormone, was found to be elevated in patients with oncological and infectious conditions and decreased post-intervention in several trials, reflecting its utility as a stress-responsive prognostic marker (47). While IL-6 has dual functions depending on the cellular context and phase of immune response, the presence of elevated levels in saliva generally reflects its pro-inflammatory role during active disease states (48). This duality does not contradict its elevation in pathological conditions, as it often predominates in a pro-inflammatory profile during acute and chronic inflammation.

### 4.2 Salivary interleukins and cortisol as prognostic markers

The interleukins most frequently reported across the included studies were IL-1β, IL-6, IL-8, TNF-α, and IL-10, all of which are central mediators of the inflammatory response. These cytokines were found to fluctuate in response to treatments such as photobiomodulation therapy (PBMT), pharmacological agents, and stress-reduction techniques. For example, IL-1β and TNF-α are typically elevated during the acute phase of inflammation, and their salivary levels were shown to decrease post-treatment in several cancer-related interventions (29–30, 38–39).

IL-6, a multifunctional cytokine with dual pro- and anti-inflammatory roles, was also widely assessed. Its regulation in saliva following oncological treatments or behavioral interventions suggests potential for monitoring immune recovery and systemic burden (32, 35, 37). Similarly, IL-8 plays a causal role in neutrophil activation and was found to correlate with disease activity and treatment response in both cancer and infectious diseases (33, 36, 42).

The inclusion of salivary cortisol in several studies highlights its role as a reliable biomarker for stress-related physiological changes. Its reduction after interventions such as cognitive behavioral stress management (CBSM), mindfulness-based stress reduction (MBSR), and yoga practices reflects its dynamic nature and prognostic significance (31–32, 35, 37, 40–41, 44).

Notably, salivary cortisol may offer real-time monitoring of hypothalamic–pituitary–adrenal (HPA) axis activity, and its immediate elevation following stress stimuli and subsequent reduction post-intervention make it a valuable marker for evaluating the systemic impact of disease and the effectiveness of therapy (49).

Several studies listed in Table 3 included nitrite as a salivary biomarker, primarily in relation to inflammatory modulation. Beyond its role as an indicator, nitrite may hold promise as a prognostic bioactive factor due to its involvement in nitric oxide metabolism, oxidative stress, and immune response regulation. Nitrite has been linked to tumor progression and therapeutic responsiveness, particularly in head and neck cancers, making it a candidate for future prognostic profiling in oncological contexts (50).

### 4.3 Differences in biomarker patterns between oncological and infectious conditions

Although both oncological and infectious diseases triggered elevated salivary cytokines and cortisol levels, the patterns of biomarker expression and their response to treatment varied depending on the underlying condition. This elevation does not imply a linear or static increase; rather, cytokine levels, particularly those like IL-6, may vary dynamically depending on disease stage, immune response, or treatment effect.

In oncological diseases, such as head and neck cancer, breast cancer, or leukemia, cytokine profiles typically reflected chronic inflammation and immune dysregulation. Interventions including PBMT, chemotherapy, or relaxation programs were associated with moderate to significant reductions in salivary IL-1β, IL-6, IL-8, and TNF-α (29–32, 35–39, 42). These reductions often coincide with clinical improvements, such as reduced oral mucositis or decreased self-reported stress. This supports the idea that salivary biomarkers may serve not only as biological indicators of inflammation but also as clinical correlates of treatment efficacy in cancer patients (2).

In contrast, infectious diseases such as HIV and tuberculosis showed a more variable pattern. For example, Rubin et al. (34) and Dudgeon et al. (43) reported acute increases in cortisol and interleukins shortly after pharmacological or physical interventions, which may reflect the immediate immune activation seen in infectious processes or early treatment responses. Similarly, Shenje et al. (36) observed complex shifts in multiple interleukins, such as IL-6 and IL-10, following corticosteroid therapy in tuberculosis patients. These findings suggest that the time course and interpretation of salivary biomarkers in infectious diseases may require more nuanced approaches and time-point–specific sampling protocols (51).

While both disease categories activate systemic immune responses, oncological patients often present with sustained elevation of biomarkers due to tumor-induced inflammation, whereas in infectious cases, fluctuations may be more rapid and transient, related to microbial load or treatment onset. These fluctuations reflect the evolving nature of immune response, where cytokine levels may rise during disease exacerbation and decline with effective treatment or resolution of infection. In oncological conditions, this modulation may follow therapeutic cycles, whereas in infections, fluctuations often correspond to microbial burden or host immune activation. Therefore, clinicians must consider disease-specific biomarker dynamics when using saliva for prognostic purposes.

### 4.4 Clinical implications and use in monitoring and follow-up

The use of salivary biomarkers such as interleukins and cortisol offers promising clinical applications in the monitoring and follow-up of patients with oncological and infectious diseases. Due to its non-invasive, painless, and low-risk nature, saliva collection is particularly suitable for patients undergoing immunosuppressive treatments, those with reduced venous access, or in pediatric and geriatric populations (48).

In clinical practice, these biomarkers could help in:

• Assessing therapeutic response, particularly in stress-reducing interventions and anti-inflammatory treatments.
• Monitoring relapse or recurrence, especially in cancers associated with persistent inflammatory responses.
• Predicting complications, such as oral mucositis or systemic immune suppression.
• Supporting psychological evaluations, where cortisol may serve as an objective stress indicator alongside patient-reported outcomes (35, 44).

Despite their potential, current evidence also reveals the absence of standardized protocols for saliva collection, processing, and biomarker quantification across studies. The included trials used different sample types (unstimulated, stimulated), kits (ELISA, cytometric bead arrays), and collection times (ranging from a few hours to several months of follow-up), which may compromise reproducibility and clinical translation.

• To overcome these challenges, future guidelines should promote the harmonization of analytical protocols, including:
• Defined time-points for sample collection (e.g., baseline, post-treatment, long-term).
• Control for circadian and environmental factors influencing cortisol and cytokines (49).
• Validated thresholds or cut-off values for specific biomarkers, distinguishing normal from pathological patterns.

With these improvements, saliva-based prognostic tools could be integrated into regular clinical assessments, enabling real-time, minimally invasive monitoring, especially in resource-limited or outpatient settings.

### 4.5 Implications for clinical practice

The findings of this review suggest that salivary biomarkers—particularly cortisol and inflammatory interleukins—may serve as useful adjuncts in the prognostic evaluation and therapeutic monitoring of patients with oncological and infectious diseases. Their non-invasive nature allows for repeated monitoring of systemic inflammation and stress-related responses, especially in vulnerable populations such as cancer patients and individuals with immunodeficiency. These biomarkers offer the potential for dynamic assessment of therapeutic effectiveness, particularly in interventions targeting inflammatory or psychological components, including corticosteroid therapy, cognitive-behavioral interventions, or photobiomodulation. Moreover, their use can improve patient compliance and comfort by avoiding the discomfort and risk associated with blood draws, especially when frequent sampling is required. In resource-limited, outpatient, or remote care settings, salivary biomarker testing may offer a practical and accessible alternative to traditional laboratory methods. Integration of salivary diagnostics into oncology clinics, infectious disease units, and primary care settings could facilitate early detection of relapse, identification of treatment-related complications, and personalized therapeutic adjustments. However, to enable their successful clinical adoption, further efforts are needed to ensure proper validation, cost-effectiveness analyses, and training for healthcare professionals in the interpretation and use salivary biomarker assays (6).

To enhance interpretability and guide future translational research, a comparative summary of the key salivary biomarkers identified in the included studies is presented in Table 5. This includes their biological role, clinical utility, limitations encountered, and suggested research directions.

Previous reviews have explored the diagnostic or prognostic role of salivary biomarkers; however, most combined diverse study designs, including observational, case-control, or *in vitro* studies, limiting the strength of their conclusions. In contrast, our systematic review is the first to synthesize only randomized controlled trials (RCTs), providing higher-level evidence on the therapeutic prognostic value of salivary biomarkers in both oncological and infectious diseases. Additionally, unlike prior reviews that were disease-specific (e.g., oral squamous cell carcinoma, COVID-19), our work provides a comparative perspective across a broader clinical spectrum. We further enhanced the methodological rigor by applying GRADE criteria to assess the certainty of evidence and by presenting a summary table that integrates biological significance, clinical utility, and future research pathways for each biomarker. These contributions offer a more robust, clinically oriented, and evidence-based foundation for future translational studies.

### 4.6 Limitations of the current evidence and of this review

Despite the strengths of this systematic review—such as the use of a registered protocol, adherence to PRISMA guidelines, and exclusive inclusion of randomized controlled trials—several limitations must be acknowledged.

First, although the studies included were RCTs, six of them presented a high risk of bias (31, 33, 37, 42–44), particularly in aspects related to blinding, deviations from intended interventions, or incomplete outcome data. This contributes to uncertainty in the overall quality of evidence, as confirmed by the GRADE assessment, which rated the certainty as low due to concerns about risk of bias and inconsistency.

The high risk of bias across several studies, especially regarding inadequate blinding and deviations from intended interventions, undermines the internal validity and may lead to overestimation or underestimation of biomarker effects. In addition, the presence of serious inconsistency—reflected in the heterogeneity of effect sizes and variability in direction of outcomes—further reduces the confidence in the aggregated results. This inconsistency is likely due to differences in study populations (oncological vs. infectious), types of interventions (pharmacological, behavioral, photobiomodulation), biomarker types (e.g., IL-1β, IL-6, cortisol, nitrite), and sample collection protocols. Without subgroup analyzes or meta-analytic synthesis, it remains difficult to disentangle whether these differences represent true biological variation or methodological artifacts. Therefore, the combination of high risk of bias and inconsistency identified in the GRADE assessment justifies downgrading the certainty of evidence and underscores the need for more robust, standardized, and homogeneous future research.

Second, the sample sizes varied widely, ranging from 14 to 322 participants, and follow-up periods were often short or inconsistently reported. This heterogeneity reduces the ability to draw firm conclusions about the duration and clinical stability of salivary biomarker changes.

Third, differences in saliva collection and analysis methods—including use of stimulated vs. unstimulated saliva, variability in ELISA kits or multiplex systems, and timing of sampling—limit cross-study comparability and may introduce methodological bias (52).

Furthermore, in patients with head and neck cancer, high-dose radiotherapy is commonly used and may lead to significant salivary gland dysfunction, including xerostomia and compositional changes in salivary secretions. These alterations can compromise the stability, concentration, and detectability of bioactive molecules, such as cytokines and cortisol, potentially limiting the reliability of salivary biomarkers for prognostic purposes in this subgroup. Consequently, the applicability of saliva-based diagnoses in irradiated populations remains a clinical and methodological challenge that warrants specific investigation (53).

Fourth, although the review included both oncological and infectious diseases, the distribution of studies was even, with a predominance of cancer-related trials. Only two studies focused on HIV (34, 43) and one on tuberculosis (36), leaving viral, bacterial, and parasitic infections underrepresented. Therefore, the generalizability of results to the broader spectrum of infectious diseases is limited.

Finally, none of the included studies evaluated longitudinal prognostic trajectories beyond 12 months, and no study reported the cost-effectiveness or feasibility of implementation of salivary testing in routine clinical care, which limits the current clinical translatability of these findings (53).

### 4.7 Implications for future research

The results of this review highlighted the potential of salivary biomarkers in the prognosis and monitoring of oncological and infectious diseases. However, the translational gap between research findings and clinical implementation remains wide, highlighting several priority areas for future research.

First, future studies should aim to validate salivary biomarkers in larger, multicenter RCTs, with adequate statistical power, standardized protocols for saliva collection and biomarker analysis, and longitudinal follow-up extending beyond 6–12 months. This will help determine whether changes in cytokine or cortisol levels are sustained over time and whether they correlate with clinical outcomes such as disease remission, relapse, or complications (54).

Second, comparative research is needed to evaluate the diagnostic and prognostic performance of salivary biomarkers vs. blood-based counterparts. If saliva proves to be equally or more sensitive, its application in non-invasive monitoring, particularly in low-resource settings or outpatient care, could be transformative (55).

Third, future investigations should include a broader spectrum of infectious diseases, such as hepatitis, COVID-19, or parasitic infections, where inflammatory and endocrine responses may be measurable in saliva. The inclusion of diverse populations, including pediatric and immunocompromised patients, will improve external validity and enhance the relevance of findings across healthcare contexts.

Fourth, attention should be given to the development of point-of-care (POC) salivary diagnostic devices, which could allow real-time monitoring of biomarker changes without requiring laboratory infrastructure. Advances in biosensors and microfluidics may soon enable rapid, portable saliva testing, revolutionizing the way clinicians track disease progression (56).

Finally, research on the cost-effectiveness, acceptability, and implementation barriers of salivary biomarker testing is urgently needed. Without this data, even highly accurate tests may fail to be adopted in clinical practice (57).

## 5 Conclusions

This systematic review indicates that salivary cortisol and interleukins, particularly IL-6, IL-8, and TNF-α, are the most frequently studied biomarkers with prognostic value in oncological and infectious diseases. Their measurable variation following interventions such as pharmacological treatments, photobiomodulation, and stress-reduction therapies supports their potential use in monitoring treatment response and disease progression. However, the heterogeneity of study designs, variability in biomarker analysis, and low certainty of evidence highlight the need for standardized protocols and further high-quality research. Future studies should focus on expanding the range of infectious conditions studied, validating salivary biomarkers against clinical outcomes, and assessing their integration into routine, non-invasive monitoring strategies.

## Data Availability

The original contributions presented in the study are included in the article/Supplementary Material.

## References

[1] Kumar P Gupta S Das BC. Saliva as a potential non-invasive liquid biopsy for early and easy diagnosis/prognosis of head and neck cancer. Transl Oncol. (2024) 40:101827. 10.1016/j.tranon.2023.101827 38042138 PMC10701368

[2] Diesch T Filippi C Fritschi N Filippi A Ritz N. Cytokines in saliva as biomarkers of oral and systemic oncological or infectious diseases: a systematic review. Cytokine. (2021) 143:155506. 10.1016/j.cyto.2021.155506 33846070

[3] Kumari S Samara M Ampadi Ramachandran R Gosh S George H Wang R A review on saliva-based health diagnostics: biomarker selection and future directions. Biomed Mater Devices. (2023) 2(1):1–18. 10.1007/s44174-023-00090-z PMC10243891 37363139

[4] Piyarathne NS Rasnayake RMSGK Angammana R Chandrasekera P Ramachandra S Weerasekera M Diagnostic salivary biomarkers in oral cancer and oral potentially malignant disorders and their relationships to risk factors: a systematic review. Expert Rev Mol Diagn. (2021) 21(8):789–807. 10.1080/14737159.2021.1944106 34148471

[5] Shirzaiy M Dalirsani Z Peymankar P Taherizadeh M. Relationship between salivary levels of interleukin-8 and HbA1c in patients with type 2 diabetes. Endocrinol Diabetes Metab. (2023) 6(6):e455. 10.1002/edm2.455 37775939 PMC10638620

[6] Mutavhatsindi H Calder B McAnda S Malherbe ST Stanley K Kidd M Identification of novel salivary candidate protein biomarkers for tuberculosis diagnosis: a preliminary biomarker discovery study. Tuberculosis (Edinb). (2021) 130:102118. 10.1016/j.tube.2021.102118 34371310

[7] Dikova VR Principe S Bagan JV. Salivary inflammatory proteins in patients with oral potentially malignant disorders. J Clin Exp Dent. (2019) 11:e659–64. 10.4317/jced.55917 31516665 PMC6731005

[8] Huang L Luo F Deng M Zhang J. The relationship between salivary cytokines and oral cancer and their diagnostic capabilities for oral cancer: a systematic review and network meta-analysis. BMC Oral Health. (2024) 24(1):1044. 10.1186/s12903-024-04840-3 39237889 PMC11378403

[9] Santosh T S Parmar R Anand H Srikanth K Saritha M. A review of salivary diagnostics and its potential implication in detection of COVID-19. Cureus. (2020) 12(4):e7708. 10.7759/cureus.7708 32313785 PMC7164701

[10] Chiappin S Antonelli G Gatti R De Palo EF. Saliva specimen: a new laboratory tool for diagnostic and basic investigation. Clin Chim Acta. (2007) 383(1–2):30–40. 10.1016/j.cca.2007.04.011 17512510

[11] Pirnia B Soleimani A Farhoudian A Zahiroddin A. Prediction of suicidal thoughts and behaviors based on the diurnal cortisol pattern and THC dosage in continued cannabis users: a 5-year population-based matched cohort study. Psychiatry Res. (2024) 339:116091. 10.1016/j.psychres.2024.116091 39068898

[12] Florezi GP Barone FP Pelissari C Soyfoo MS Delporte C Lourenço SV. Salivary Th17-associated cytokines as potential biomarkers in primary sjögren’s disease. Oral Surg Oral Med Oral Pathol Oral Radiol. (2025) 140(4):428–35. 10.1016/j.oooo.2025.04.171 40450455

[13] Chen X Aqrawi LA Utheim TP Tashbayev B Utheim ØA Reppe S Elevated cytokine levels in tears and saliva of patients with primary sjögren’s syndrome correlate with clinical ocular and oral manifestations. Sci Rep. (2019) 9:7319. 10.1038/s41598-019-43714-5 31086200 PMC6513950

[14] Almeslet A Alnamlah S Alanzan L Aldriwesh R AlWehaiby S. Role of salivary biomarkers in cystic fibrosis: a systematic review. Biomed Res Int. (2022) 2022:5818840. 10.1155/2022/5818840 35097122 PMC8791744

[15] Corral MA T Daza EH Jimenez NA Morales Vera DZ Velosa Porras J Latorre Uriza C Biomarkers for the severity of periodontal disease in patients with obstructive sleep apnea: iL-1β, IL-6, IL-17A, and IL-33. Heliyon. (2023) 9(3):e14340. 10.1016/j.heliyon.2023.e14340 36967976 PMC10031375

[16] Nejstgaard CH Sondrup N Chan AW Dwan K Moher D Page MJ A scoping review identifies comments suggesting modifications to PRISMA-P 2015. J Clin Epidemiol. (2025) 182:111760. 10.1016/j.jclinepi.2025.111760 40107391

[17] Pieper D Rombey T. Where to prospectively register a systematic review. Syst Rev. (2022) 11(1):8. 10.1186/s13643-021-01877-1 34998432 PMC8742923

[18] Sterne JAC Savović J Page MJ Elbers RG Blencowe NS Boutron I Rob 2: a revised tool for assessing risk of bias in randomized trials. Br Med J. (2019) 366:l4898. 10.1136/bmj.l4898 31462531

[19] Nandan SRK Kulkarni PG. Salivary tumor necrosis factor-α as a biomarker in oral leukoplakia and oral squamous cell carcinoma. Asian Pac J Cancer Prev. (2019) 20:2087–93. 10.31557/APJCP.2019.20.7.2087 31350970 PMC6745219

[20] Carvalho MFS Cavalieri D Do Nascimento S Lourenco TGB Ramos DVR Pasqualin DC Cytokines levels and salivary microbiome play a potential role in oral lichen planus diagnosis. Sci Rep. (2019) 9:19037. 10.1038/s41598-019-54615-y 31792433 PMC6889227

[21] Jacobs R Tshehla E Malherbe S Kriel M Loxton AG Stanley K Host biomarkers detected in saliva show promise as markers for the diagnosis of pulmonary tuberculosis disease and monitoring of the response to tuberculosis treatment. Cytokine. (2016) 81:50–6. 10.1016/j.cyto.2016.02.004 26878648

[22] Pels E. Comparison of saliva interleukin-2 concentration to the condition of gums in children with acute lymphoblastic leukaemia during anti-tumor treatment. Cancer Chemother Pharmacol. (2015) 76:205–10. 10.1007/s00280-015-2750-7 25976216 PMC4485694

[23] Sharma M Bairy I Pai K Satyamoorthy K Prasad S Berkovitz B Salivary IL-6 levels in oral leukoplakia with dysplasia and its clinical relevance to tobacco habits and periodontitis. Clin Oral Investig. (2011) 15:705–14. 10.1007/s00784-010-0435 20563615

[24] Carlson LE Speca M Faris P Patel KD. One year pre-post intervention follow-up of psychological, immune, endocrine and blood pressure outcomes of mindfulness-based stress reduction (MBSR) in breast and prostate cancer outpatients. Brain Behav Immun. (2007) 21:1038–49. 10.1016/j.bbi.2007.04.002 17521871

[25] Small EJ Carducci MA Burke JM Rodriguez R Fong L van Ummersen L A phase I trial of intravenous CG7870, a replication-selective, prostate-specific antigen-targeted oncolytic adenovirus, for the treatment of hormone-refractory, metastatic prostate cancer. Mol Ther. (2006) 14:107–17. 10.1016/j.ymthe.2006.02.011 16690359

[26] Rhodus NL Cheng B Myers S Miller L Ho V Ondrey F. The feasibility of monitoring NF-*κ*B associated cytokines: tNF-α, IL-1*α*, IL-6, and IL-8 in whole saliva for the malignant transformation of oral lichen planus. Mol Carcinog. (2005) 44:77–82. 10.1002/mc.20113 16075467

[27] Abdel Fattah Tarrad N Gamil Shaker O Abdelkawy M Hassan S. Association of serum and salivary dipeptidyl peptidase-4 (DPP-4) with oral cancerous and precancerous lesions: an observational diagnostic accuracy study. BMC Oral Health. (2024) 24:1206. 10.1186/s12903-024-04939-7 39390508 PMC11468375

[28] Ucciferri C Falasca K Reale M Tamburro M Auricchio A Vignale F Pidotimod and immunological activation in individuals infected with HIV. Curr HIV Res. (2021) 19:260–8. 10.2174/1570162X18666210111102046 33430735

[29] Pereira CH Martins AFL Morais MO de Sousa-Neto SS da Silva ACG Arantes DAC Oral mucositis management with photobiomodulation, Bidens pilosa L. (Asteraceae) and Curcuma longa L. (Zingiberaceae), the FITOPROT herbal medicine, and its influence on inflammatory cytokine levels: a randomized clinical trial. Support Care Cancer. (2024) 32:628. 10.1007/s00520-024-08842-3 39223301

[30] Martins AFL Morais MO Sousa-Neto SS Oton-Leite AF Pereira CH Valadares MC The effect of photobiomodulation on nitrite and inflammatory activity in radiotherapy-induced oral mucositis: a randomized clinical trial. Lasers Surg Med. (2021) 53:671–83. 10.1002/lsm.23328 32997817

[31] Hoyt MA Wang AW Breen EC Nelson CJ. A randomized controlled trial of goal-focused emotion-regulation therapy for young adult survivors of testicular cancer: effects on salivary and inflammatory stress markers. Am J Mens Health. (2021) 15:15579883211044556. 10.1177/15579883211044557 PMC8436315 34514890

[32] Penedo FJ Fox RS Walsh EA Yanez B Miller GE Oswald LB Effects of web-based cognitive behavioral stress management and health promotion interventions on neuroendocrine and inflammatory markers in men with advanced prostate cancer: a randomized controlled trial. Brain Behav Immun. (2021) 95:168–77. 10.1016/j.bbi.2021.03.014 33737170 PMC8888023

[33] Basak S Bera A Yoon A Morselli M Jeong C Tosevska A A randomized, phase 1, placebo-controlled trial of APG-157 in oral cancer demonstrates systemic absorption and an inhibitory effect on cytokines and tumor-associated microbes. Cancer. (2020) 126:1668–82. 10.1002/cncr.32644 32022261

[34] Rubin L Langenecker S Phan K Keating S Neigh G Weber K Remitted depression and cognition in HIV: the role of cortisol and inflammation. Psychoneuroendocrinology. (2020) 114:104609. 10.1016/j.psyneuen.2020.104609 32062371 PMC7254879

[35] Lengacher C Reich R Paterson C Shelton M Shivers S Ramesar S A large randomized trial: effects of mindfulness-based stress reduction (MBSR) for breast cancer (BC) survivors on salivary cortisol and IL-6. Biol Res Nurs. (2019) 21:39–49. 10.1177/1099800418789777 30079756 PMC6700883

[36] Shenje J Lai RP Ross IL Mayosi BM Wilkinson RJ Ntsekhe M Effect of prednisolone on inflammatory markers in pericardial tuberculosis: a pilot study. IJC Heart Vasc. (2018) 18:104–8. 10.1016/j.ijcha.2017.10.002 PMC5941241 29750184

[37] Campo R Light K O’Connor K Nakamura Y Lipschitz D LaStayo P Blood pressure, salivary cortisol, and inflammatory cytokine outcomes in senior female cancer survivors enrolled in a tai chi chih randomized controlled trial. J Cancer Survival. (2015) 9:115–25. 10.1007/s11764-014-0395-x PMC4344390 25164513

[38] Oton-Leite A Silva G Morais M Silva T Leles C Valadares M Effect of low-level laser therapy on chemoradiotherapy-induced oral mucositis and salivary inflammatory mediators in head and neck cancer patients. Lasers Surg Med. (2015) 47:296–305. 10.1002/lsm.22349 25824475

[39] Silva G Sacono N Othon-Leite A Mendonça E Arantes A Bariani C Effect of low-level laser therapy on inflammatory mediator release during chemotherapy-induced oral mucositis: a randomized preliminary study. Lasers Med Sci. (2015) 30:117–26. 10.1007/s10103-014-1624-2 25037968

[40] Bower J Greendale G Crosswell A Garet D Sternlieb B Ganz P Yoga reduces inflammatory signaling in fatigued breast cancer survivors: a randomized controlled trial. Psychoneuroendocrinology. (2014) 43:20–9. 10.1016/j.psyneuen.2014.01.019 24703167 PMC4060606

[41] Saxton JM Scott E Daley AJ Woodroofe MN Mutrie N Crank H Effects of an exercise and hypocaloric healthy eating intervention on indices of psychological health status, hypothalamic-pituitary-adrenal axis regulation and immune function after early-stage breast cancer: a randomized controlled trial. Breast Cancer Res. (2014) 16:R39. 10.1186/bcr3643 24731917 PMC4052984

[42] Morales-Rojas T Viera N Morón-Medina A Alvarez CJ Alvarez A. Proinflammatory cytokines during the initial phase of oral mucositis in patients with acute lymphoblastic leukaemia. Int J Paediatr Dent. (2012) 22:191–6. 10.1111/j.1365-263X.2011.01175.x 21919984

[43] Dudgeon W Phillips K Durstine J Burgess S Lyerly G Davis J Individual exercise sessions alter circulating hormones and cytokines in HIV-infected men. Appl Physiol Nutr Metab. (2010) 35:560–8. 10.1139/H10-045 20725124

[44] Nelson EL Wenzel L Osann K Dogan-Ates A Chantana N Reina-Patton A Stress, immunity, and cervical cancer: biobehavioral outcomes of a randomized clinical trial. Clin Cancer Res. (2008) 14:2111–8. 10.1158/1078-0432.CCR-07-1632 18381952 PMC4572837

[45] Khurshid Z Zafar MS Khan RS Najeeb S Slowey PD Rehman IU. Role of salivary biomarkers in oral cancer detection. Adv Clin Chem. (2018) 86:23–70. 10.1016/bs.acc.2018.05.002 30144841

[46] Wang X Kaczor-Urbanowicz KE Wong DTW. Salivary biomarkers in cancer detection. Med Oncol. (2017) 34:7. 10.1007/s12032-016-0863-4 27943101 PMC5534214

[47] Song M Bai H Zhang P Zhou X Ying B. Promising applications of human-derived saliva biomarker testing in clinical diagnostics. Int J Oral Sci. (2023) 15(1):2. 10.1038/s41368-022-00209-w 36596771 PMC9810734

[48] Dongiovanni P Meroni M Casati S Goldoni R Thomaz DV Kehr NS Salivary biomarkers: novel noninvasive tools to diagnose chronic inflammation. Int J Oral Sci. (2023) 15(1):27. 10.1038/s41368-023-00231-6 37386003 PMC10310701

[49] Joseph NT Jiang Y Zilioli S. Momentary emotions and salivary cortisol: a systematic review and meta-analysis of ecological momentary assessment studies. Neurosci Biobehav Rev. (2021) 125:365–79. 10.1016/j.neubiorev.2021.02.042 33662445

[50] Gallo O Masini E Morbidelli L Franchi A Fini-Storchi I Vergari WA Role of nitric oxide in angiogenesis and tumor progression in head and neck cancer. J Natl Cancer Inst. (1998) 90(8):587–96. 10.1093/jnci/90.8.587 9554441

[51] Constantin V Luchian I Goriuc A Budala DG Bida FC Cojocaru C Salivary biomarkers identification: advances in standard and emerging technologies. Oral (Basel). (2025) 5(2):26. 10.3390/oral5020026

[52] Liao C Chen X Fu Y. Salivary analysis: an emerging paradigm for non-invasive healthcare diagnosis and monitoring. Interdiscip Med. (2023) 1(3):1–20. 10.1002/inmd.20230009

[53] Li Y Ou Y Fan K Liu G. Salivary diagnostics: opportunities and challenges. Theranostics. (2024) 14(18):6969–90. 10.7150/thno.100600 39629130 PMC11610148

[54] Ross RE VanDerwerker CJ Saladin ME Gregory CM. The role of exercise in the treatment of depression: biological underpinnings and clinical outcomes. Mol Psychiatry. (2023) 28(1):298–328. 10.1038/s41380-022-01819-w 36253441 PMC9969795

[55] Pittman TW Decsi DB Punyadeera C Henry CS. Saliva-based microfluidic point-of-care diagnostic. Theranostics. (2023) 13(3):1091–108. 10.7150/thno.78872 36793864 PMC9925318

[56] Wasilewski T Kamysz W Gębicki J. AI-assisted detection of biomarkers by sensors and biosensors for early diagnosis and monitoring. Biosensors (Basel). (2024) 14(7):356. 10.3390/bios14070356 39056632 PMC11274923

[57] Cirillo N. A roadmap for the rational use of biomarkers in oral disease screening. Biomolecules. (2024) 14(7):787. 10.3390/biom14070787 39062501 PMC11274832

